# Ultrastructure of the Interlamellar Membranes of the Nacre of the Bivalve *Pteria hirundo*, Determined by Immunolabelling

**DOI:** 10.1371/journal.pone.0122934

**Published:** 2015-04-24

**Authors:** Antonio J. Osuna-Mascaró, Teresa Cruz-Bustos, Frédéric Marin, Antonio G. Checa

**Affiliations:** 1 Departamento de Estratigrafía y Paleontología, Facultad de Ciencias, Universidad de Granada, Av. Fuentenueva, S/N 18071, Granada, Spain; 2 Departamento de Bioquímica y Parasitología Molecular, Facultad de Ciencias, Universidad de Granada, Av. Fuentenueva, S/N 18071, Granada, Spain; 3 UMR CNRS 6282 Biogéosciences, Université de Bourgogne, 6 Bd. Gabriel, 21000, Dijon, France; University of California, UNITED STATES

## Abstract

The current model for the ultrastructure of the interlamellar membranes of molluscan nacre imply that they consist of a core of aligned chitin fibers surrounded on both sides by acidic proteins. This model was based on observations taken on previously demineralized shells, where the original structure had disappeared. Despite other earlier claims, no direct observations exist in which the different components can be unequivocally discriminated. We have applied different labeling protocols on non-demineralized nacreous shells of the bivalve *Pteria*. With this method, we have revealed the disposition and nature of the different fibers of the interlamellar membranes that can be observed on the surface of the nacreous shell of the bivalve *Pteria hirundo* by high resolution scanning electron microscopy (SEM). The minor chitin component consists of very thin fibers with a high aspect ratio and which are seemingly disoriented. Each fiber has a protein coat, which probably forms a complex with the chitin. The chitin-protein-complex fibers are embedded in an additional proteinaceous matrix. This is the first time in which the sizes, positions and distribution of the chitin fibers have been observed in situ.

## Introduction

The shell of molluscs is a biocomposite of calcium carbonate (aragonite, calcite or, very rarely, vaterite) and a small quantity of organic matrix in the form of proteins, polysaccharides and lipids [1]. The proportion of organic matrix varies in the different microstructures, with the crossed lamellar being the poorest (1%), and nacre, columnar prismatic (calcitic and aragonitic) and granular prismatic (>7%) being increasingly richer [2–6]

Chitin, the second most abundant natural polymer after cellulose, is a fundamental structural component in the shells of molluscs. Its extraordinary evolutionary success is due to its ability to combine with proteins and other compounds to form strong hybrid materials. The presence of chitin in the shells of molluscs (cephalopods) was first reported by Meyer in 1913 [7], and later by many other authors [8], where it forms complexes with the protein [2,9]. Hackman [10] and Okafor [11] determined the *ϐ*-nature of the chitin in *Sepia*. Regarding nacre, the presence of chitin in the cephalopod *Nautilus macromphalus* was first reported by Goffinet [12] by using a chitinase treatment. A better understanding of the ultrastructure of the organic fraction of nacre was obtained with the combined results of Weiner & Traub (1980) [13] and Weiner et al. (1983) [14] with X-ray and electron diffraction, respectively. These authors showed that the intercrystalline matrix of the three mayor classes of molluscs is composed of silk-fibroin proteins and the *ϐ*-polymorph of chitin. They also concluded that the protein sheets and the chitin fibers are well aligned and that the *a*-axes of the aragonite platelets align with the chitin *b*-axis. The same applies to the aragonite *b*- and *c*-axes with respect to the corresponding axes of the protein which implied that the crystal orientation upon nucleation is governed, either directly or indirectly by the organic matrix (heterohepitaxial growth model) [14,15].

By using cryo-TEM, Levi-Kalisman [16], proposed a model for the ultrastructure of the interlamellar membranes (ILMs) of the nacre of the pinnid bivalve *Atrina serrata*, which is widely accepted today as being general for nacre [17][18–21]. According to this model, the ILMs consist of a core of highly ordered fibers of *ϐ*-chitin arranged in planes and surrounded on both sides by acidic proteins. Levi-Kalisman [16] established their model of parallel orientation based on both their results and those of Weiner [13,14]. In their study, Levi-Kalisman [16] identified chitin in demineralized shells, from which suspended pellets were obtained; something that inevitably leads to the loss of the original distribution of the components of the ILMs.

Subsequent SEM observations of glutaraldehyde-fixed and non-demineralized samples of bivalves and gastropods, preserving the original relation of the structural components, show a felt-like arrangement of disordered fibers interwoven in the plane of the ILM [8,22–26]. The main difference between bivalves and gastropods is the existence of multiple pores in the spaces between fibers in the ILMs of the latter.

Schäffer [18], Nudelman [19] and Bezares [20,27] made similar observations, either with AFM or SEM or both, on samples treated with proteinase K. Much of the ILM volume disappeared and the residual ultrastructure consisted of a network of thin fibers, which were densely distributed and locally amalgamated. Nudelman [19] in addition, observed that such network disappeared after chitinase treatment, which led them to conclude that they formed the entire chitin core of the ILMs. Bezares [20] used WGA-labeling for chitin and found a strong marking in the intertabular membranes, but not in the ILMs. Despite this, they attributed a chitinous nature to the fibrous meshwork. This view was maintained in Bezares [21,27,28]. Schäffer [18] were uncertain as to the purely chitinous nature of the fibers, since they were aware that some protein is still present after protease treatment. This is also expressed in Bezares et al. (2012) [28], who refers to the fibrous structures previously interpreted as chitin in Bezares et al. (2008) [20] to be “in fact protein and not chitin” (their p. 351). Our own data (see below) also imply that removing entirely the protein fraction with proteinase K in order to expose pure chitin is in fact impossible. Additionally, after prolonged treatment, the whole network is lost, which suggests that after complete removal of the protein, the chitin framework is not self supported. Nudelman’s [19] results indicate that chitin is present after protease treatment, but, given the small proportion of chitin within the ILMs of nacre [29], it cannot be concluded that the fibrous network they observed represents the actual morphology of the chitin network (their images also show evident changes in the density of the material).

Identifying beyond any doubt the position and morphology of the chitin components of the ILMs is essential for several reasons. First, the parallel-fiber model of Levi-Kalisman [16] is difficult to reconcile with the SEM and AFM observations that the fibers composing the core form a felt-like arrangement (see above), unless what is observed does not correspond to the actual chitin distribution.

Cartwright & Checa [24] proposed that the formation of the ILMs of nacre proceeds by liquid crystallization. In their view, chitin fibers arrange themselves according to the cholesteric phase of a liquid crystal. Ideally, this is a layered structure, with the fibers or molecules arranged perfectly parallel within each plane, but slightly rotated with respect to those of the adjacent planes, so that after a certain distance or ‘pitch’ the orientation is exactly the same. In this way, a spiral arrangement is finally obtained. Cartwright & Checa [19] observed the disordered nature of the fibers, which they inferred to be composed of chitin. They subsequently proposed that the liquid crystalline cholesteric phase is made of chitin fibers distributed into layers but disordered within each layer. Shortly after, the chitin-fiber layers are coated with protein, which prevents further co-orientation. Subsequently, the growth of aragonite platelets takes place at the spaces between interlamellar layers. Cartwright & Checa [24] assumed, but did not demonstrate the chitinous nature of the fibers.

In order to check whether the fibers which have been observed under SEM constitute the chitin fraction of the ILMs, we have applied labelling protocols to unaltered (i.e. non-demineralized) nacre of the glassy wing-oyster *Pteria hirundo*. To perform a labeling that can be observed and analyzed in SEM, the most appropriate technique is the immunogold. For immunogold, usually the sample is incubated with a specific antibody designed to bind the target of interest. Then, a secondary antibody (or protein A) which has gold particles attached is added, and it binds to the primary antibody. Gold particles can be easily seen in SEM and, with the appropriate treatment and different sizes of gold particles, it can be used for labelling different targets. Our results have provided essential insight as to the ultrastructure and a more accurate view of the ILMs of bivalve nacre.

## Materials and Methods

No specific permissions were required for this work. *Pteria hirundo* is not an endangered species and it was collected from excess fisheries.

### Material

Specimens of the pterioidean bivalve *Pteria hirundo* were collected directly from the coast of Málaga (SE Spain), and immersed *in vivo* in 70% ethanol, thereby avoiding the acidification process which could damage the inner surface of the shell if the specimens were transferred alive to the laboratory (personal observations). They were then stored in a refrigerator at 4°C until processing. An empty shell of the abalone *Haliotis rufescens* (unknown location, California, USA), was also used.

### Preliminary sample preparation

For confocal microscopy, large fragments of the *Haliotis* shell were cut using a water-refrigerated cold saw. Smaller, ~2x2 cm, samples were later cut out using a Multi-Dremel rotating disc. Each sample was previously fixed to a microscopy slide and then polished using carborundum (silicon carbide, SiC) with water. The grit size was reduced sequentially from 80 to 320. Samples were polished until they reached a thickness of 80–100 μm, taking care that they had no apparent flaws or scratches under the optical microscope.

The two valves of the shells of *Pteria hirundo* were separated and the animal's soft parts were removed, taking care to minimize the damage to the shell. The valves were washed repeatedly in running water in order to remove any traces of ethanol. For immunogold, we cut fragments ~1x1 cm of the nacreous internal shell surface with pliers (in order to prevent heating of the sample), trying to avoid the non-nacreous myostracal area. Minor, particle-like fragments also used for immunogold were obtained by scraping the internal nacre surface of the shell with a blade.

### Confocal microscopy

*Haliotis* samples polished and fixed to microscopy slides were treated with proteinase K (0.02 g/ml) and incubated with wheat germ agglutinin(WGA, Sigma) and fluorescein isothiocyanate (FITC, Sigma), in darkness and at constant humidity for 30 min.

Control samples were treated with chitinase (1 mg/ml) in citrate buffer (0.5 M, pH 5.5) for 2 h at 50°C. Samples were mounted using 70% glycerol in PBS, and observed in a Leica DMI6000 inverted microscope. Fluorescein isothiocyanate has excitation and emission spectrum peak wavelengths of approximately 495 nm/519 nm.

### Inmunogold treatment

Given the particularities of the experiment, WGA has been used prior to the first antibody and protein A attached to gold particles, instead of the second antibody. Gold can be attached to protein A instead of a secondary antibody, this protein bind antibodies in a non-specific way. For SEM labelling, were used two complementary methods of immunogold, one to label chitin and other for protein.

In order to expose the presumed chitin fibers, we tried to gently eliminate the protein coating with different concentrations of proteinase K (from 0.01 to 0.2 g/ml) in order to find an optimum concentration. A first control (C1) consisting of untreated samples was established. Treatments lasted for 2 hours in oven at 55°C. Five washes of 5 minutes each in distilled water, and in exactly the same manner in PBS, followed.

At this stage, a second control (C2), in which the samples were additionally treated with chitinase (1 mg/ml) in citrate buffer (0.5 M, pH 5.5), for 2 h at 50°C, in order to remove chitin, was implemented. Five washes in PBS, of five minutes each, followed.

Experimental samples were later blocked for 13 min with filtered gelatin (to prevent non-specific binding of antibodies). We used PBS OVA to remove the gelatin. Subsequently, the samples were exposed to WGA for either 16h at 4°C or 2h at 30°C, and washed with PBS, 5 times (per 5 min each). They were then incubated with an antibody which is specific for lectin (Anti-WGA, Sigma), diluted 1/20, from 1 to 3 h, at 30°C. The remains of the anti-lectin antibody were removed with 5 washes (per 5 minutes) with PBS-OVA.

Two additional subsets of samples were chosen as controls: a subset untreated with anti-lectin antibody (for possible non-specific binding) (C3a) and another subset without both proteinase K and antibody (to assess for non-specific binding to protein) (C3b).

The experimental samples were finally incubated with protein A bounded to colloidal gold (20 nm thick particles), for 3h at 30°C. Samples were finally washed with PBS and water 5 times (x 5 minutes), and dried thereafter in an oven at 35°C.

### Treatment of particulate samples for immunogold

The particles were divided into several tubes and treated as the fractured samples, however during each wash the sample had to be centrifuged to settle the particles on the bottom of the tube.

### Double immunogold

For double immunogold, samples were first subjected to the same treatment as above for experimental immunogold samples. For additional protein labeling, we used a pool of bivalve nacre antibodies (prepared by FM) consisting of: k508def7, antibody elicited against acetic acid-soluble matrix (ASM) of the nacre of *Pinctada margaritifera*, K5089 and k5090, elicited against the nacre ASM of the *Pinna nobilis*, and K4952 elicited against the nacre ASM of *Bathymodiolus* sp. The three species are of molluscan bivalves, being *P*. *margaritifera* and *P*. *nobilis* being closely allied to *P*. *hirundo*. Some of these antibodies have a strong cross reactivity with the ASM of the nacre of the latter species. The pool was incubated overnight in a dilution 1/20 at 4°C. Samples were washed 5 times (x 5 min) with PBS and then incubated with anti-rabbit antibodies labeled with 10-nm thick colloidal gold particles for 2 h at 37°C. Finally, the samples were washed with both PBS and water, and oven-dried.

### SEM

Once dried, the samples were mounted on aluminium stubs and carbon coated (Hitachi UHS evaporator). The observations were performed in the field emission SEM (FESEM) Zeiss Auriga Cross-Beam Station of the Centro de Instrumentación Científica (CIC) of the University of Granada. The best conditions for observation of the ILMs and associated gold particles were obtained in secondary electron (SE), In-Lens and energy selective backscattered (ESB) modes, at 3 kV.

## Results

### Confocal microscopy

**Fig 1:**
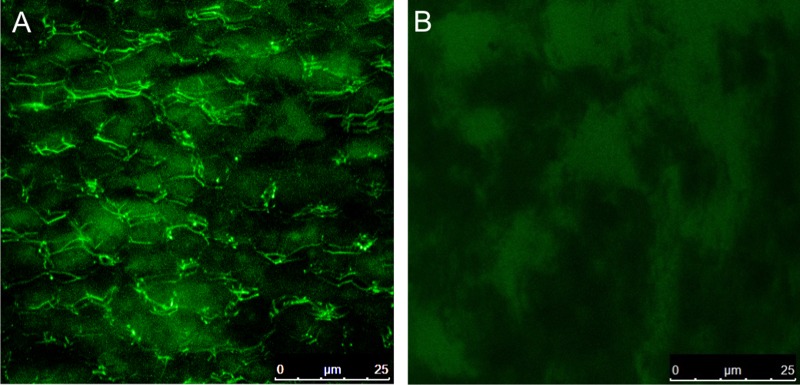
Confocal microscopy of *Haliotis* sp. nacre samples treated with WGA bound to fluorescein isothiocyanate (FITC). A. Experimental sample, with strong fluorescence at the contacts between plates (intertabular membranes). B. Control sample, in which no signal is observed.

Samples treated with WGA bound to fluorescein isothiocyanate (FITC) showed a strong fluorescence at the contacts between plates, while no signal was observed in the control samples (Fig 1).

### Scanning Electron Microscopy

#### Non-treated samples

**Fig 2:**
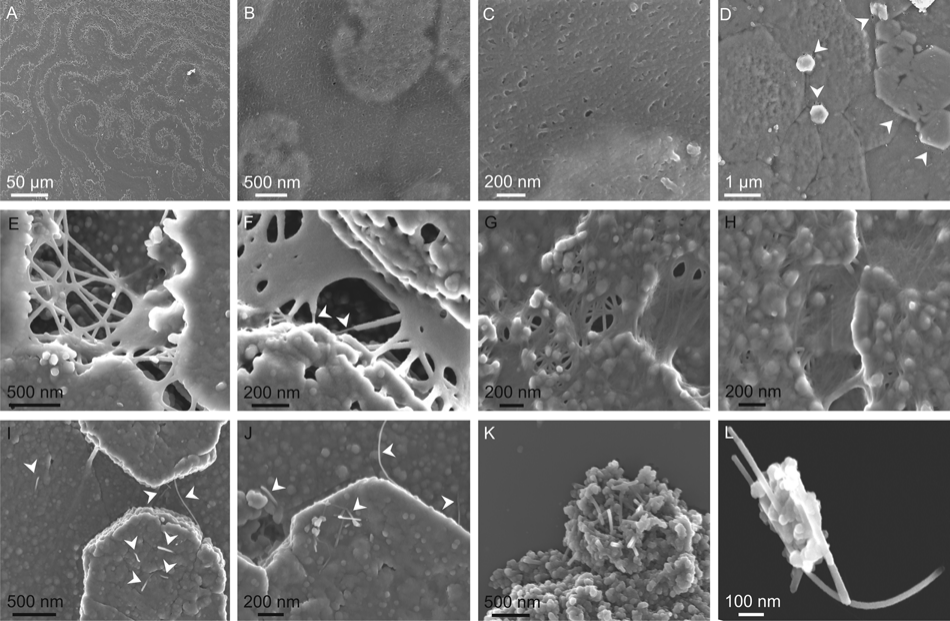
Untreated nacre samples. A. General aspect of the nacre. The spiral and fingerprint-like pattern of nacre growth fronts are particularly well developed. B, C. Untreated specimen in which the last formed ILM extends horizontal between the growing plates (seen by transparency). The fibrous aspect of the ILM is particularly evident in the close up view (C). D. Specimen in which the last-formed ILM has stuck onto the previous lamella around the edges of the plates (arrows). E, F. Frayed portions of ILMs subtending from platelets, showing the formation of type 1 fibers. Some of these fibers continue into type 2 fibers in F. G, H. Same situation as in E and F, in which, in addition, type 2 fibers slightly stand out from the ILMs. I, J. Type 2 fibers sprouting from nacre platelets (arrows). K, L. Nacre particles obtained by scraping and containing type 2 fibers.

**Fig 3:**
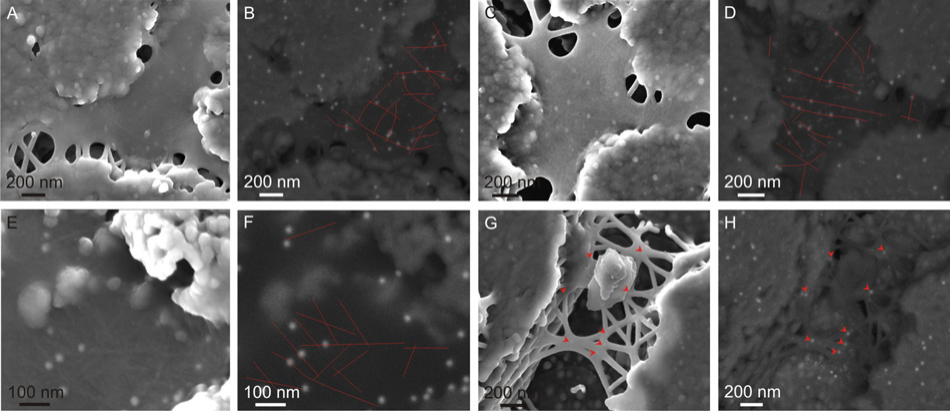
Secondary electron (SE, left) and backscattered electron (BSE, right) paired images of membranes labelled for protein (small lucent dots in the BSE, right images,) and chitin (big dots). A-F. The tracings of several fibers of type 2 are evident (some of them have been outlined in red in the BSE images). Most of the big particles fall onto any of such fibers. Some labeling appears in the upper right tablet, despite the fact that the ILM is absent on it. G-H. Frayed membrane. The junctions between type 1 fibers are frequently the sites of deposition of big particles.

The samples of *Pteria hirundo* without treatment showed the spiral, labyrinthine and target-like patterns typical of the nacre of bivalves, which result from the shape of the growth fronts of the terraced tablet lamellae (Fig 2A). The thin ILMs cover the platelets and subtend across the spaces between immature platelets, where they can retain the same aspect as on the platelets (Fig 2B and 2C) or appear frayed upon contraction (Fig 2D–2H), something which is enhanced by the dehydrating action of the alcohol. When membranes take the latter aspect, we can observe two types of fibers: Type 1 fibers are thick (*ca*. 40–60 nm in average) and with the appearance of having been stretched, they form a meshwork-like structure as local continuations of the membrane (Figs 2E–2G and 3G). They are wider at the ends, where they connect with other fibers, than at the center, thus reflecting a slightly viscous behavior. Type 2 fibers are thinner (between 5 and 15 nm thick) and more uniform in thickness (Figs 2G, 2H, 3A, 3C and 3E); they are usually straight, although some may appear gently and uniformly curved. Most usually, they are embedded within the membrane, being recognizable because they slightly protrude from the otherwise smooth surface of the membrane. Some of the fibers can be followed for up to ~1 μm (Figs 2G, 2H and 3G). Given the small extensions of the subtending parts of the ILMs and the difficulty of recognizing the embedded type 2 fibers, these have to be considered minimal lengths. Favorable observation conditions (Figs 2G, 2H, 3A, 3C and 3E) show the high density of type 2 fibers, which is more evident the closer the view (Fig 3E). Occasionally, type 1 fibers become thinner laterally and transform into fibers of type 2 (Fig 2F). Type 2 fibers are commonly observed extending beyond the platelets, where they particularly have the appearance of being stiffer than those of type 1 (Fig 2I and 2J), as well as of the shell particles obtained by scraping (Fig 2K and 2L).

#### Inmunolabeled experimental samples

From the wide range of concentrations of proteinase K, the best results for both simple and double immunolabeling were obtained at a concentration of 0.02 g/ml. At higher concentrations (0.05 g/ml), the degree of labeling decreased drastically, above which all the interlamelar membranes were gone, although scattered 20 nm gold particles remained over the platelets. Extremely high concentrations of proteinase K (0.1–0.2 g/ml) led to the development of the "hourglass" patterns within the nacre platelets first described by Mutvei [30] and later observed and interpreted by Checa [31].

With the double labeling, the ILMs (both in the areas which both extend freely and on the platelets) appear studded with tiny gold particles (10 nm) used for labeling proteins (Fig 3). The disorganized and rigid type 2 fibers that occur embedded within the membrane appear covered with 20 nm gold particles intended for labeling chitin. This is best observed where the ILMs subtend between platelets, because it is here where the embedded fibers of type 2 stand out due to the lack of any substrate with might deform the topography of the flat ILMs (Fig 3A–3F). In general where the type 2 fibers can be recognized without a doubt, the 20 nm gold particles lie exactly on or, when there is more than one on the same fiber, align strictly with the fiber. In a few instances, 20 nm gold particles cannot be associated to type 2 fibers, which does not imply the inexistence of such fibers, given the difficulty inherent to their recognition. Labeling also occurs on those areas of the ILMs extending on the surfaces of the platelets, but the irregularity of the underlying granular platelet surface largely hinders the recognition of corresponding fiber (Fig 3A–3H). The intersections between fibers of type 1 (where the ILM shows a meshwork-like structure) commonly appear labeled for chitin (20 nm gold particles) (Fig 3G and 3H). Rare instances of 20 nm particles have been observed on platelets from above which the ILM had disappeared (Fig 3E and 3F).

The intracrystalline fibers protruding from some platelets and shell particles (Fig 2I–2L) did not show any kind of labeling.

#### Immunolabeled control samples

**Fig 4:**
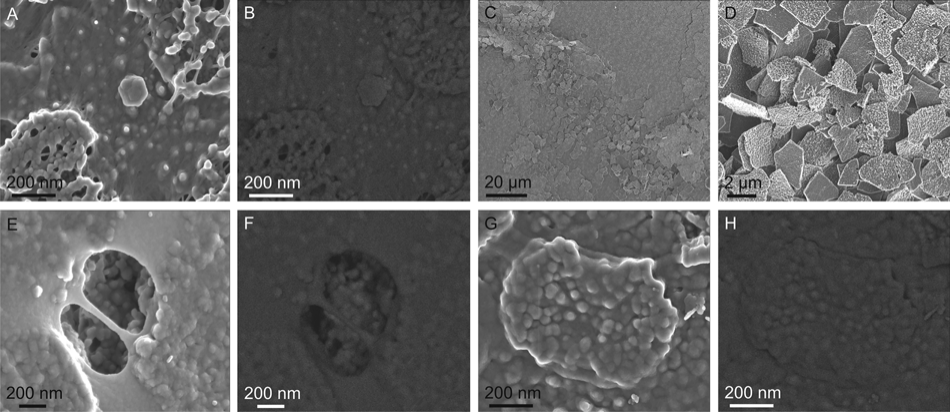
Control samples. A, B. Control C1. Secondary electron (SE, left) and backscattered electron (BSE, right) views of a labeled sample not treated with proteinase K. No labelling is evident in the BSE image. C, D. Control C2. Samples treated with chitinase. The nacre platelets appear superficially corroded and dislodged. E, F. Control C3a. SE (left) and BSE (right) views of a sample treated without anti-lectin antibody. The BSE image shows the absence of labelling. G, H. Control C3b. SE (left) and BSE (right) views of a sample treated without both anti-lectin antibody and proteinase K. No labelling appears in the BSE image.

C1. The controls without proteinase K showed no labeling for chitin (Fig 4A and 4B), which suggests that either chitin is not present or it is covered by protein.

C2. In samples treated with chitinase, the nacre platelets appeared slightly damaged and completely dislodged and disorganized (Fig 4C and 4D), as if, literally, they had lost their fixation. ILMs, as well as 20 nm gold particles are totally absent. The corrosion observed may partly due to the action of the citrate buffer used (pH 5.5).

C3. The samples treated without anti-lectin antibody of any of the following two controls, C3a, without antibody, and C3b, without both antibody and proteinase K, showed neither specific (as expected for this type of control), nor nonspecific binding (Fig 4E–4H).

## Discussion

As expected, the best immunolabeling results have been obtained on experimental samples labeled for both chitin and protein. Particularly in the areas where ILMs subtend between growing platelets, where type 2 fibers stand out from the background membrane, there are very consistent marks for chitin (20 nm particles), while the interspaces and the type 1 fibers are labeled for protein (10 nm particles) (Fig 3). The lack of labeling in our control samples treated without anti-lectin (C3a) (Fig 4E and 4F) and without both anti-lectin antibody and proteinase K (C3b) (Fig 4G and 4H) shows that binding is specific for chitin, and, thus, reveals the reliability of our techique. Labeling is also absent in the controls without proteinase K (C1) (Fig 4A and 4B), which implies that a partial removal of the protein is necessary to expose them to the binding activity of the lectins. Labeling for chitin drastically decreased with higher concentrations of proteinase K. We infer that above a certain degree of removal of the protein, the type 2 fibers, which are intimately related to the protein, begin to become freed and lost in the solution, until the complete removal of the ILM.

In conclusion, our different immunolocation techniques allow us to deduce that the nature of the fibers observed by FESEM in the nacre surface of the bivalve *Pteria hirundo* is twofold: big fibers (type 1) linking platelets as an extension of the ILMs are mainly composed of protein, whereas thin rigid fibers (type 2) (most usually observed embedded within the membrane) are of chitin. Chitin fibers are prevalent in the ILMs of *Pteria hirundo*, although their contribution to the bulk volume is difficult to estimate. Although these fibers are different from the thick (type 1) proteinaceous fibers, the latter may also consist of a chitin nucleus. This is proven by the existence of type 2 fibers apparently emerging from type 1 fibers (Fig 2F), which suggests that the latter result when, upon contraction or pulling of the ILM, the chitin network becomes partly frayed, but the chitin fibers still retain a protein coating. In other instances, type 1 fibers may be entirely made of protein (e.g., Fig 2E and 2F). There are also type 2-like fibers which protrude from within the platelets (Fig 2I and 2J) and from the particles obtained by scraping (Fig 2K and 2L). We have not succeeded in labeling them for chitin, possibly due to their rareness, but bearing in mind their apparent stiffness and resemblance in dimensions with the chitin-labeled type 2 fibers of the ILMs, we suggest that they are chitin too. The fact that some 20 nm gold particles remained over the platelets (Fig 3E and 3F), might be due to the presence of relatively occluded intracrystalline type 2 fibers, of the kind observed with SEM. This would imply that there is an as yet undetected intracrystalline chitin fraction. Chitin is also present in the intertabular membranes of the nacre of *Haliotis*, according to the immunolabelling study of Bezares [20] and to our confocal microscopy study (Fig 1). Therefore, chitin is ubiquitous in the different components of nacre.

Our study demonstrates beyond any doubt that chitin (shown to be *ϐ*-chitin by Weiner & Traub [13]) is an essential component of the ILMs. Nevertheless, attempts to completely remove the protein in order to expose the actual aspect of the chitin framework have proved unsuccessful. This is most likely due to the fact both components form complexes [32], for which some models exist in arthropods [33]. The estimates of the percent weight of the chitin compared to the whole organic fraction are highly variable, but with values always <7% [20,29,34]. Interestingly, the lowest values have been reported by Goffinet [29] for two species of *Pinctada* (*P*. *margaritifera*, 0.17%; *P*. *galtsoffi*, 0.19%), which is a genus of the family Pteriidae, to which our experimental species, *Pteria hirundo*, also belongs.

Given these figures, it is clear that the felt-like frameworks, which previous authors implied were the chitin cores of the ILMs of related bivalves [20] and *Haliotis* [21,28], must have contained high amounts of protein. Accordingly, conclusions about the degree of alignment of the fibers [28] on protease-treated ILMs have to be taken with caution, because we can never be sure up to which point the observed residual framework reflects the distribution of the chitin framework. On the other hand, the AFM images of similar fibers of Schäffer [18] and Bezares [20], with diameters of 5–10 nm, accord better with what can be expected for chitin, based on the comparison with the type 2 fibers we have observed. The measured low proportions of chitin within the framework explain why our prolonged treatments with proteinase completely removes the ILMs (see above) and why additional chitinase treatment leads to nacre disorganization (Fig 4C and 4D). What is not clear is why only chitinase leads to the complete elimination of the ILMs in the two bivalves studied by Nudelman [19], one of them being also a Pteriidae.

## Conclusion

**Fig 5:**
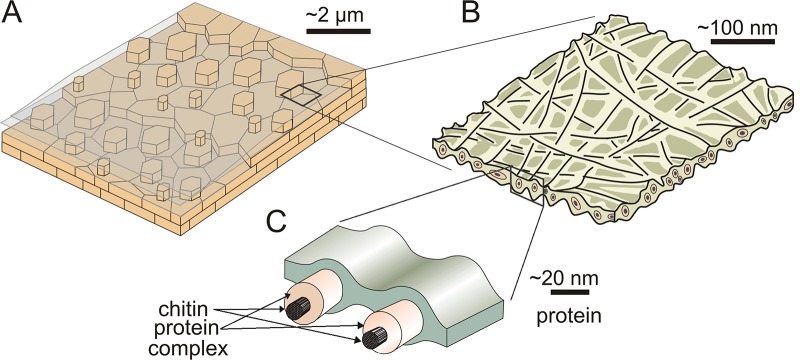
Sketch depicting the ultrastructure of the ILMs of nacre. A. General structure of the nacre of bivalves. Platelets arrange into terraces and the ILMs extend on top of the last formed lamella, beyond the growth of the lamella. B. General structure of an ILM. It has a fibrous aspect, with the long fibres seemingly disoriented and extending within the plane of the membrane. C. Detail of the ultrastructure of the ILM. It is composed by protein fibers which surround a chitin core fiber, thus forming a chitin-protein complex. The network is embedded in an additional protein matrix.

The ILMs of nacre consist of very thin fibres of *ϐ*-chitin (our type 2 fibers) coated by protein (our type 1 fibers), which is covalently linked to the chitin, thus forming complexes. According to the aspect of untreated ILMs, this framework must be embedded in an additional proteinaceous matrix. A simple ultrastructural model is shown in Fig 5.

There is nevertheless much yet to be known about the ultrastructure of the ILMs, such as the exact diameters and lengths of, and relationships (distribution, alignment and interweaving) between the chitin fibers.

The most widely accepted model for the structure of the ILMs of nacre was proposed by Levi-Kalisman [16], according to which, chitin fibers are densely distributed and highly ordered within the ILMs. Our new evidence on *Pteria hirundo*, as well as the previous evidence on other groups (mainly bivalves and gastropods), indicates that the chitin fibers are consistently disorganized in the plane of the ILM. Accordingly, levi-kalisman’s [16] model has to be reconsidered.

Cartwright and Checa [24] took this disorganization into consideration when they proposed their formation of ILMs by means of liquid crystallization (see above). According to this model, the chitin frameworks of successive ILMs become self-organized within the extrapallial space according to a layer-by-layer liquid crystal. If during the initial stage of chitin layering, a chitin fiber is not added to either the underlying or overlying ILMs, it will remain in the space between two ILMs where it will finally be either absorbed by a growing nacre platelet, thus leading to an intracrystalline chitin fiber, or trapped within the intertabular membranes.

One of the appeals of the study of nacre is that it is still an open question, thus giving full validity to the assertion of the statistician George E. P. Box [35]: “Remember that all models are wrong; the practical question is how wrong do they have to be to not be useful”.
